# Advance in therapies targeting tumor-associated macrophages in ovarian cancer

**DOI:** 10.3389/fimmu.2025.1677839

**Published:** 2025-09-11

**Authors:** Man Li, Yue Ma, Tinggeng Dai, Yongxin Wang, Ying Yue

**Affiliations:** Department of Gynecological Oncology, The First Hospital of Jilin University, Changchun, China

**Keywords:** ovarian cancer, tumor-associated macrophages, tumor microenvironment, epithelial-mesenchymal transition, chemoresistance, TAM polarization

## Abstract

Ovarian cancer remains the deadliest gynecologic malignancy, with its aggressive progression and therapeutic resistance heavily influenced by the tumor microenvironment (TME). Tumor-associated macrophages (TAMs), the predominant immune infiltrates in OC, play pivotal roles in metastasis, immunosuppression, and chemoresistance by adopting a pro-tumoral M2 phenotype. Despite promising preclinical results, clinical translation faces challenges, such as on-target toxicity and incomplete understanding of TAM ontogeny in humans. This review summarizes the origins, heterogeneity, and functional plasticity of TAMs, emphasizing their mechanistic contributions to OC progression through epithelial-mesenchymal transition (EMT), angiogenesis, and immune evasion. We outline the emerging evidence that TAMs drive platinum resistance via exosomal signaling and metabolic reprogramming, underscoring TAMs as central mediators of OC pathogenesis and treatment paradigms.

## 1 Introduction

Ovarian cancer remains the most lethal gynecologic malignancy worldwide, with its pathogenesis and progression intricately shaped by the tumor microenvironment (TME)—a dynamic ecosystem comprising not only malignant epithelial cells, but also adipocytes, vasculature, stromal fibroblasts, lymphocytes, dendritic cells, cancer-associated fibroblasts, and tumor-associated macrophages (TAMs) (1, 2). Through continuous bidirectional communication with both cellular and acellular components, tumor cells operate as adaptive entities that integrate cues from immune, endocrine, and nervous systems to construct a self-sustaining niche promoting oncogenesis, metastasis, and therapeutic resistance (3). The ovarian cancer TME is notably immunosuppressive, fostering unchecked tumor expansion and evasion of host surveillance (4, 5).

Among immune components of the ovarian cancer TME, macrophages constitute the predominant infiltrating population (6, 7). These cells contribute to multiple hallmarks of malignancy, including facilitating intravasation of tumor cells into the circulation and suppressing anti-tumor immunity. Fibroblasts represent another critical population, supporting migration of tumor cells from the primary site, aiding systemic dissemination, and guiding endothelial cells during tumor angiogenesis (8). Increasing evidence highlights TAMs as central mediators of bidirectional crosstalk between tumor cells and the TME. In ovarian cancer, TAMs predominantly exhibit an immunosuppressive M2 phenotype that promotes tumor growth, invasion, angiogenesis, immune evasion, and metastatic competence (9). Given these multifaceted roles, TAMs have emerged as key targets for therapeutic intervention. This review summarizes the mechanistic roles of TAMs in ovarian cancer progression and explores strategies to modulate TAM function, with the aim of identifying innovative approaches to improve outcomes for patients with ovarian cancer.

## 2 Origins of TAMs and emerging concepts

Macrophages were once thought to arise exclusively from circulating monocytes (10). However, lineage-tracing studies have challenged this notion, revealing that while many macrophages originate from bone marrow and splenic progenitors, a considerable proportion are established during embryogenesis and persist as self-renewing tissue-resident macrophages (TRMs) (11, 12). During development, macrophages derived from the yolk sac and fetal liver seed peripheral tissues and are later complemented by bone marrow–derived monocytes in response to injury, infection, or inflammation (13). These insights have reshaped the traditional M1/M2 polarization paradigm. For instance, TAMs expressing CD163 or CD206—markers typically aligned with M2 phenotypes—can exhibit M1-like, T cell–activating properties in gastrointestinal cancers and ovarian cancer ascites (12). M1/M2 dichotomy oversimplifies the functional continuum of macrophages in the TME (14). High-dimensional analyses, including single-cell RNA sequencing, have revealed remarkable heterogeneity that transcends classical classifications. In tumors, TAMs predominantly exhibit M2-like features, though a minority display M1-like traits, contributing to tumor initiation, angiogenesis, and metastasis. Human TRMs lack definitive lineage markers, leaving their developmental origins and specialized roles insufficiently characterized (12). This underscores an urgent need to clarify macrophage heterogeneity and lineage diversity in human tumors to advance precision immunotherapy.

## 3 Roles of TAMs in ovarian cancer

### 3.1 TAMs facilitate ovarian cancer metastasis

TAMs, the predominant immune cell population within the ovarian tumor microenvironment (TME), are critical mediators of tumor progression and metastatic dissemination (15). These cells facilitate tumor cell proliferation, invasion, and the establishment of peritoneal metastases, processes closely linked to malignant ascites, recurrence, and poor prognosis. Within ascitic fluid, tumor cells frequently aggregate into multicellular spheroids that adhere to peritoneal mesothelium, initiating secondary lesions (16). Tos et al (17). revealed that highly metastatic ovarian cancer cells, upon intrabursal injection in mice, produce persistent peritoneal dissemination, whereas non-metastatic lines fail to spread. Mechanistically, β-catenin signaling, which underpins tumor growth and invasion, plays a key role; its silencing reduces omental metastases and metastatic nodules, with concurrent depletion of CD68^+^ and CD163^+^ TAMs (7, 18). β-catenin activation in tumor cells upregulates EMT-promoting transcription factors such as ZEB1 and Snail, as well as chemokines including CCL2 and CCL3, which recruit monocytes and polarize them into M2-like TAMs (17, 19–22). These recruited TAMs secrete high levels of CCL2 and IL-6, which act on tumor cells via CCR2 and IL-6R, respectively (23, 24). IL-6 binding activates the JAK/STAT3 axis, driving expression of EMT-related genes and enhancing tumor motility (25). Simultaneously, CCL2–CCR2 signaling stimulates NF-κB activity, which synergizes with β-catenin to reinforce EMT programs (26). This cytokine-driven positive feedback loop sustains mesenchymal states, promotes invasion and peritoneal dissemination, and perpetuates TAM recruitment and polarization. The EMT program further amplifies CCL2 production, fostering continuous macrophage influx and establishing a self-reinforcing TAM–tumor interaction that accelerates migration, invasion, and metastasis (27).

### 3.2 TAMs promote chemoresistance in ovarian cancer

Cytoreductive surgery followed by platinum-based chemotherapy remains the standard treatment for ovarian cancer. However, resistance to platinum compounds—whether intrinsic or acquired—remains a central challenge in disease management, often driven by pre-existing resistant clones or selective pressure from repeated treatments (28). Accumulating evidence implicates the TME as a major contributor to relapse and drug resistance (29). TAMs are predominant and contribute to angiogenesis, immune evasion, metastasis, and particularly chemoresistance (30). Although initially characterized in breast cancer, TAM-driven resistance is increasingly recognized in ovarian cancer (31). Notably, the M2-polarized subset of TAMs is closely associated with tumor progression, immune evasion, and drug resistance (32). Li et al (30). reported elevated serum circITGB6 in patients with platinum-resistant ovarian cancer compared to platinum-sensitive cases, which was accompanied by an expansion of M2 macrophages, suggesting circITGB6-driven M2 polarization as a mechanism of resistance (30). Similarly, Jang et al (33). reported that co-culture of ovarian cancer cells with macrophages reduced carboplatin sensitivity in a dose-dependent manner, coinciding with a shift toward an M2-like phenotype. Importantly, emerging preclinical evidence supports the therapeutic potential of inhibiting exosome secretion to counter TAM-mediated chemoresistance (34, 35). For example, the pharmacological inhibitor GW4869, which blocks neutral sphingomyelinase and thereby suppresses exosome biogenesis and release, has been shown to reduce exosomal miR-223 levels, restore PTEN expression, and enhance cisplatin sensitivity in ovarian cancer cells co-cultured with TAMs (35–37). *In vivo*, GW4869 administration attenuates tumor growth and enhances chemotherapy efficacy, further validating exosome-targeted interventions as a promising strategy to disrupt TAM–tumor crosstalk and overcome drug resistance in ovarian cancer (38). Zhu et al (37). further demonstrated that hypoxic ovarian cancer cells recruit macrophages and polarize them into a TAM-like phenotype, whose exosomal miR-223 confers chemoresistance *in vitro* and *in vivo* through the PTEN–PI3K/AKT pathway.

## 4 Therapeutic targeting of TAMs in ovarian cancer

### 4.1 Depletion of TAMs

The folate receptor beta (FRβ) is specifically overexpressed on M2-polarized TAMs in various epithelial malignancies, including ovarian cancer, making it a promising immunotherapeutic target for modulating the tumor microenvironment (39). Unlike FRα, which is mainly expressed on tumor cells, FRβ localizes predominantly on immune cells within the TME, particularly immunosuppressive macrophages (40, 41). In syngeneic mouse models, chimeric antigen receptor (CAR) T cells engineered to recognize FRβ have demonstrated the ability to selectively eradicate FRβ^+^ TAMs (42). This approach effectively reshapes the immunosuppressive TME into a pro-inflammatory milieu, enhancing monocyte influx, endogenous CD8^+^ T cell recruitment, delaying tumor progression, and prolonging survival (43). In syngeneic mouse models, chimeric antigen receptor (CAR) T cells directed against FRβ selectively eradicated FRβ^+^ TAMs, thereby reshaping the tumor microenvironment toward a pro-inflammatory state, with enhanced monocyte influx, recruitment of endogenous CD8^+^ T cells, delayed tumor progression, and prolonged survival. Rodriguez-Garcia et al (42). further demonstrated that murine FRβ CAR-T treatment led to transient weight loss but specific depletion of FRβ^+^ TAMs and conferred a significant survival advantage.

In contrast, the folate receptor alpha (FRα) is overexpressed directly on tumor cells in epithelial ovarian cancer, and its selective expression pattern has enabled the development of antibody–drug conjugates (ADCs) for targeted tumor cell elimination (44, 45). Mirvetuximab soravtansine (MIRV) is an FRα-targeting ADC that has demonstrated clinical efficacy in platinum-resistant epithelial ovarian cancer (46). In the phase II SORAYA trial, Matulonis et al (44). evaluated MIRV in FRα-high, platinum-resistant epithelial ovarian cancer previously treated with bevacizumab (48% ≥3 prior lines, 13% prior PARP inhibitors). MIRV monotherapy yielded a high objective response rate (ORR) with durable responses and a favorable safety profile, irrespective of prior therapies. Subsequently, Moore et al (47). in a phase III trial compared MIRV to standard chemotherapy and reported lower rates of grade ≥3 adverse events, fewer dose reductions, and discontinuations. In patients with high FRα expression, MIRV surpassed chemotherapy on secondary endpoints, showing higher ORR, greater CA125 responses, and improved patient-reported outcomes, although progression-free survival did not differ significantly. MIRV has now been approved for FRα-positive, platinum-resistant epithelial ovarian, fallopian tube, or primary peritoneal cancers after one to three prior regimens (48). The ADC therapeutic field is rapidly expanding, with >20 ongoing trials across gynecologic malignancies, including phase III (NCT04296890) of MIRV in FRα-high platinum-resistant ovarian cancers (49, 50).

### 4.2 Reduction of TAM recruitment

**Figure 1:**
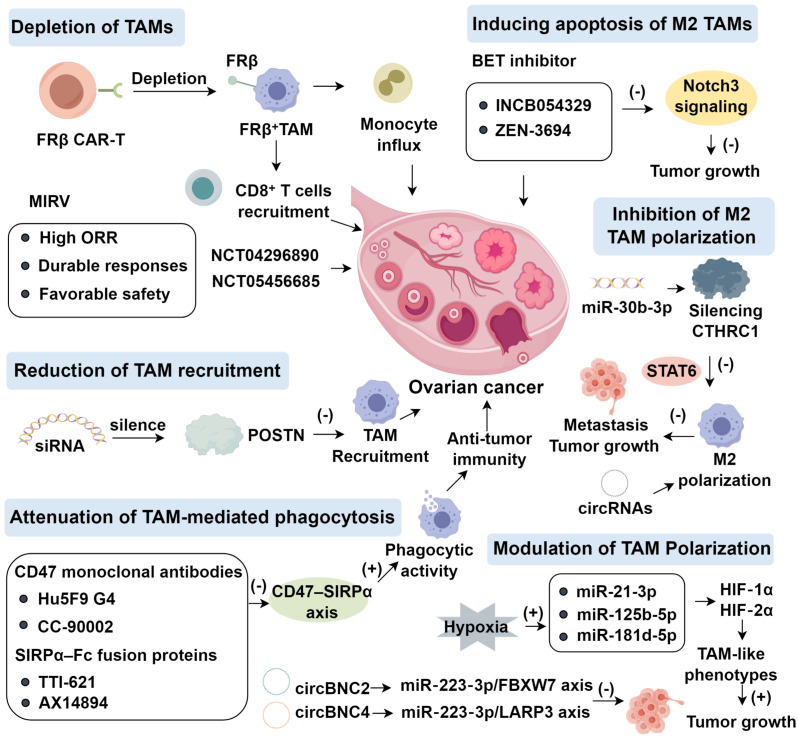
Therapeutic Targeting of TAMs in Ovarian Cancer.

Periostin (POSTN), a secreted matricellular protein, is implicated in tumor progression and poor prognosis across diverse malignancies, including ovarian cancer (51). POSTN overexpression enhances migration, chemoresistance, and macrophage recruitment (52). Tang et al (53). demonstrated that siRNA-mediated POSTN silencing in A2780 ovarian cancer cells markedly diminished chemotactic recruitment of THP-1–derived or M-CSF–induced macrophages toward A2780-conditioned medium (CM). Similarly, Zeng et al (54). showed that POSTN depletion from intrahepatic cholangiocarcinoma CM suppressed macrophage migration, whereas POSTN supplementation restored monocyte invasion. POSTN strongly attracts macrophages and drives M2 polarization, identifying it as both a prognostic biomarker and therapeutic target. Lin et al (51). reported that POSTN enrichment in invasive ovarian cancer correlates with increased migration, invasion, and metastasis, whereas knockdown reduced tumor growth *in vivo*. Mechanistically, POSTN activates integrin–FAK/NF-κB signaling, inducing cytokines (MIP-1β, MCP-1, TNF-α, RANTES), thereby enhancing monocyte chemotaxis and M2 polarization (55); metastases from POSTN-overexpressing SKOV3 cells were enriched in cancer-associated fibroblasts (CAFs) (51). DDR2-expressing CAFs regulate POSTN via ITGB1 to activate PI3K/AKT and Src pathways (56). Furthermore, LINC00520 upregulates POSTN by sponging miR-577, triggering ILK/Akt/mTOR activation; POSTN knockdown or ILK/Akt/mTOR inhibition (OSU-T315) abrogates these effects (57). Beyond oncology, POSTN deficiency exacerbates alcohol-associated liver disease in mice, whereas hepatic POSTN restoration is protective (58). Despite its central role, no clinical study has yet targeted POSTN directly (**Figure 1**).

### 4.3 Attenuation of TAM-mediated phagocytosis

CD47 is a widely expressed glycoprotein that transmits a “don’t eat me” signal through interaction with signal regulatory protein α (SIRPα) on macrophages, a mechanism exploited by tumor cells to evade immune surveillance (59). Blocking CD47–SIRPα interactions restores phagocytic activity and has emerged as a key immunotherapeutic strategy. Inhibiting CD47–SIRPα signaling reinstates macrophage phagocytic function and represents a promising immunotherapeutic approach (60, 61). Therapeutic candidates include anti-CD47 monoclonal antibodies Hu5F9 G4 and CC-90002, and SIRPα–Fc fusion proteins such as TTI-621 and ALX14894 (62–64). In a phase Ib trial (NCT02953782), Hu5F9-G4 combined with cetuximab yielded encouraging responses in advanced solid tumors, including late-stage ovarian cancer (65). In Sézary syndrome, CD47 expression is upregulated by interleukins 4 (66). Blocking CD47–SIRPα with the decoy receptor TTI-621 enhances macrophage phagocytosis and reduces tumor burden. A phase I trial reported by Ansell et al. (67) confirmed the safety and clinical responses of TTI-621 monotherapy in various hematologic malignancies, including B- and T-cell lymphomas. Mechanistically, TTI-621 not only augments macrophage function but also enhances CD8^+^ T cell cytotoxicity and promotes M1 polarization in synergy with anti–PD-L1, effectively suppressing lymphoma growth *in vitro* (68). Beyond enhancing phagocytosis, ALX148 activates dendritic cells and reprograms TAMs toward an inflammatory phenotype, thereby stimulating innate antitumor immunity. Evorpacept (ALX78), a next-generation fusion protein comprising a modified SIRPα D1 domain linked to an inactive human IgG1 Fc fragment with half the molecular weight of a conventional antibody, represents another CD47-targeted approach (69). Lakhani et al. (41) demonstrated that evorpacept is hematologically safe and, in preclinical models, synergizes with anti–PD-1/PD-L1 antibodies to enhance phagocytosis, pro-inflammatory polarization, dendritic cell activation, and cytotoxic immune responses. A phase II study (NCT05467670) is testing ALX148 with liposomal doxorubicin and pembrolizumab in platinum-resistant ovarian cancer. Ligufalimab (AK117), a novel humanized IgG4 anti-CD47 antibody, binds CD47 with high affinity while avoiding hemagglutination (70).

### 4.4 Inducing apoptosis of M2 TAMs and reprogramming toward an M1-like phenotype

Bromodomain and extraterminal domain inhibitors (BETi), which regulate epigenetic transcription by binding bromodomains, have emerged as promising modulators of TAMs. In ovarian cancer, Wilson et al (71). reported that the BET inhibitor INCB054329 impairs homologous recombination and augments the efficacy of poly (ADP-ribose) polymerase inhibitors (PARPis), whose clinical benefit is limited by resistance and toxicity. Novel delivery platforms, including JQ1-loaded nanocarriers, further improve outcomes. In ovarian and breast cancer models, Juan et al (72). showed that JQ1-loaded formulations enhanced antiproliferative effects and synergized with olaparib, while Villar-Prados et al (73). demonstrated that BET inhibition suppresses Notch3 signaling and reduces tumor growth *in situ*. ZEN-3694, a BET inhibitor, is currently under clinical evaluation in combination regimens for solid tumors, including recurrent ovarian cancer (NCT05422794, NCT05327010, NCT03901469, NCT04986423, NCT04471974, NCT05071937) (74).

Beyond direct anti-tumor effects, recent evidence suggests that BET inhibitors also modulate the tumor immune microenvironment by reprogramming tumor-associated macrophages (75). BET inhibition downregulates M2-polarizing transcription factors such as IRF4 and STAT6, while simultaneously enhancing NF-κB–dependent pro-inflammatory gene expression, thereby promoting a phenotypic switch from immunosuppressive M2 macrophages to antitumor M1 macrophages (76, 77). This reprogramming leads to increased secretion of cytokines like IL-12 and TNF-α, enhanced antigen presentation, and improved cytotoxic T cell recruitment (78, 79). Notably, in breast and ovarian cancer models, JQ1 treatment reduced macrophage infiltration and upregulated MHC II and iNOS expression in macrophages, supporting M1 polarization and fostering an inflamed TME conducive to immune-mediated tumor clearance (80, 81). In addition, BETi-mediated epigenetic remodeling suppresses the expression of immune checkpoint molecules such as PD-L1 on both tumor cells and TAMs, potentially enhancing the efficacy of checkpoint inhibitors (82–84). These dual effects on tumor cells and immune components underscore the therapeutic promise of BET inhibitors as both direct anti-cancer agents and immunomodulators. In parallel, M1 macrophage-derived extracellular vesicles (M1 MEVs) have been proposed to reprogram TAMs. Schweer et al (85). demonstrated that human M1 MEVs robustly induce M2-to-M1 repolarization both in isolated macrophages and in co-culture with ovarian cancer cells, and can target tumor xenografts, although clinical translation remains unproven.

### 4.5 Inhibition of M2 TAM polarization

M2-polarized TAMs, as dominant components of the TME, critically drive migration, invasion, immune evasion, and therapeutic resistance in ovarian cancer. In epithelial ovarian cancer, overexpression of CTHRC1 promotes EMT, thereby enhancing tumor invasion and metastasis (86), a mechanism also implicated in lung, gastrointestinal, breast, and pancreatic cancers (87). Ovarian cancer cells secrete CTHRC1, which activates STAT6 signaling in TAMs, inducing their M2 polarization. These M2 TAMs, in turn, further stimulate tumor migration and invasion, forming a positive feedback loop. Silencing CTHRC1 abrogates STAT6-mediated M2 polarization, suppresses metastasis, and delays disease progression, highlighting CTHRC1 as a potential therapeutic target. Additionally, miR-30b-3p, downregulated in ovarian cancer R3 cells, suppresses proliferation, promotes apoptosis, slows cell cycle progression, and inhibits migration and invasion upon overexpression; it directly targets CTHRC1, thereby linking it to EMT and suggesting its potential as a biomarker and therapeutic candidate (88). Circular RNAs (circRNAs) provide an additional regulatory layer. circITGB6 interacts with IGF2BP2 and FGF9 mRNA to stabilize FGF9 transcripts, induce M2 polarization, and confer cisplatin resistance (14). Combined cisplatin and antisense oligonucleotide (ASO) targeting circITGB6 markedly suppress tumor growth and improve survival.

### 4.6 Modulation of TAM polarization

Hypoxia, a hallmark of solid tumors, profoundly shapes ovarian cancer progression (51). In ascitic fluid, exosomal miR-940 is transferred to macrophages, reprogramming them toward an M2 phenotype that promotes ovarian cancer cell proliferation and migration (89). Thus, miR-940 functions as a tumor-promoting regulator through TAM polarization. In parallel, hypoxic stress elevates the levels of miR-21-3p, miR-125b-5p, and miR-181d-5p in ovarian cancer–derived exosomes. Uptake of these vesicles by macrophages which are mediated via HIF-1α and HIF-2α induces TAM-like phenotypes that further enhance tumor growth and metastatic potential (90). Importantly, inhibition of miR-223 partially attenuates TAM-derived exosome–induced chemoresistance, indicating that additional exosomal cargos, including proteins and other miRNAs, contribute to drug resistance (37). Among these, the miR-223/PTEN/PI3K/AKT axis has been identified as a major driver of chemoresistance in ovarian cancer cells, underscoring exosomes as potential therapeutic targets to restore chemosensitivity. Recent findings demonstrated that circ-BNC2 inhibits ovarian cancer progression via the miR-223-3p/FBXW7 axis (91). FBXW7, a recognized tumor suppressor, inhibits EMT in oral squamous cell carcinoma through PI3K/AKT signaling (92) and regulates proliferation and apoptosis in colorectal cancer via Notch and Akt/mTOR pathways (93). In ovarian cancer, FBXW7 expression is reduced and inversely associated with miR-223-3p while positively correlating with circ-BNC2, and it functionally suppresses invasion and migration (94). Moreover, circ-BNC4/miR-223-3p/LARP3 axis was identified with similar regulatory implications (95).

## 5 Conclusion

The immunosuppressive tumor microenvironment (TME) in ovarian cancer fosters immune evasion, metastasis, and chemoresistance. Tumor-associated macrophages (TAMs), predominantly M2-polarized, play central roles in these processes. Accordingly, therapeutic strategies targeting TAMs—through depletion, recruitment blockade, phagocytosis restoration, apoptosis induction, or phenotype reprogramming—offer promising avenues. However, translational barriers remain, including TAM heterogeneity, lack of specific markers, incomplete understanding of macrophage ontogeny in humans, and potential on-target toxicity. Most TAM-targeted therapies are still in early-phase trials without definitive clinical validation.

To bridge these gaps, future studies should employ single-cell and spatial transcriptomics to define TAM subsets, develop humanized models that recapitulate the TME, and design rational combination therapies. Biomarker-guided clinical trials are essential to optimize patient selection and therapeutic efficacy. In sum, a deeper mechanistic understanding of TAM plasticity and intercellular networks will be key to advancing TAM-directed interventions toward clinical translation in ovarian cancer.
